# The Correlation between Lower Extremity Fracture and Subsequent Arterial Embolism and Thrombosis—A National Population Cohort Study

**DOI:** 10.3390/jcm10225312

**Published:** 2021-11-15

**Authors:** Jian-Xun Chen, Shao-Yun Hsu, Mei-Chen Lin, Pin-Keng Shih

**Affiliations:** 1 School of Medicine, China Medical University, Taichung 404, Taiwan; joseph.jx.chen@gmail.com (J.-X.C.); coolindm@gmail.com (M.-C.L.); 2 Department of Surgery, China Medical University Hospital, Taichung 404, Taiwan; 3 Division of Reconstructive Microsurgery, Department of Plastic and Reconstructive Surgery, Chang Gung Memorial Hospital, College of Medicine, Chang Gung University, Taoyuan 333, Taiwan; fishy9681@gmail.com; 4 Management Office for Health Data, China Medical University and Hospital, Taichung 404, Taiwan

**Keywords:** arterial embolism and thrombosis, lower leg fractures

## Abstract

The hazard of subsequent arterial embolism and thrombosis (SAET) in patients with lower leg fractures is not yet well demonstrated. The purpose of this study is to determine the correlation between lower leg fracture and SAET in Taiwan. A total of 134,844 patients with lower leg fractures (ICD-9-CM: 823) and chronological diagnosis as SAET (ICD-9-CM: 444.22) was matched (1:1) to the non-fracture cohort according to their propensity score (data coming from the National Health Insurance database between January 2000 to December 2012). Patients were matched by age, gender, and comorbidities. The incidence of SAET and correlation between SAET development and lower leg fracture was statistically analyzed, and subgroup analysis categorized by characteristics and comorbidities was conducted as well. The cumulative incidence of SAET was calculated by Kaplan–Meier analysis. Kaplan–Meier analysis plot showed that, by the end of the ten-year follow-up period, the cumulative incidence of SAET was significantly higher for the lower leg fracture cohort than for the non-fracture cohort (log-rank test: *p* < 0.001). The lower leg fracture, male, elder age (45–64-year-old; ≥65-year-old), hypertension, diabetes mellitus, and gout were significantly associated with lower extremity SAET risk compared with the matched group. There was an inseparable correlation between the lower leg fracture group and the risks of SAET; subgroup analysis by gender (male, female), age (age < 40 years, age 40–64 years, and age > 65 years) and comorbidities (hypertension, diabetes mellitus, and gout) show compatible results as well. Patients with lower leg fracture have a significantly increased risk of SAET since then two years after the fracture. The hazard of SAET was significantly higher in patients with lower leg fracture than in the non-fracture cohort, and the high incidence was found since then two years after fracture. Further studies are warranted.

## 1. Introduction

Emerging evidence discloses that the lower leg fractures cause bone malalignment, soft tissue injury, and impairment of adjacent vessel patency [1,2,3]. Patients with vascular damage usually present with tibia-fibula, open and mid-shaft fractures [4]. The anterior tibial artery was the most commonly damaged vessel and manifested as complete arterial occlusion [5]. Artery insufficiency resulting from vessel damage may present either as acute limb ischemia or as non-union fracture or osteomyelitis in long-term follow-up [6,7]. Nevertheless, the riskiest period of vessel impairment remains unknown. Therefore, routine angiographic survey after lower leg fracture remained a controversy.

The lower leg fractures associated with peripheral artery occlusion may result from mechanical or biochemical factors [2]. Posttraumatic pseudoaneurysm with subsequent leg ischemia was the most common mechanical cause [8,9]. Regarding the biochemical elements, the thrombin–antithrombin complex and tissue factors increase considerably after fractures. [10]. Additionally, inflammatory factors, such as tumor necrosis factor-alpha, interleukin-6, D-dipolymer, and platelet count remarkably elevated after legs fractured. Hypercoagulative status are common inpatient with femoral shaft fracture who requires low-dose aspirin (<100 μg/mL) or low molecular weight heparin therapies [11,12].

Presently, few studies demonstrate the correlation between lower leg fractures and subsequent arterial embolism and thrombosis (SAET). Our research is designed to study this issue and determine the chronological incidence, risk factors, parameters of SAET after lower leg fracture.

## 2. Materials and Methods

### 2.1. Data Source

The National Health Insurance Program has enrolled nearly 99% of the Taiwan population and documented their medical record in the National Health Insurance Research Database (NHIRD) since 1995. The database included medical visits of the clinic, emergency department, hospitalization, operations, and any medical service of every medical institution in Taiwan. This study was conducted by population-based hospitalization file. All individuals with at least one hospitalization for corresponding diagnosis identification number are included. The diagnoses identification numbers are defined based on the International Classification of Disease, Ninth Revision, Clinical Modification (ICD-9-CM). The Research Ethics Committee of China Medical University and Hospital in Taiwan approved this study (CMUH-104-REC2-115-(AR4)).

### 2.2. Study Population

To clarify the correlation between lower leg fracture and the risk of SAET, we enrolled all the patients with tibia and fibula fracture (ICD-9-CM: 823) from 2000 to 2012 as one study cohort; without tibia and fibula fracture as the comparing cohort. Patients in both cohorts were followed up until 31 December 2013. The index date of enrolling SAET was set on the date when the database records a diagnosis of the tibia and fibula fracture (ICD-9-CM: 823). Patients diagnosed with lower extremity SAET (ICD-9-CM: 444.22) and peripheral vascular disease (ICD-9-CM: 443.9) before the index date or aged < 18 (calculated until the index date) were excluded from this study.

Considering that previous studies have a well-document that patients with hip trauma are at risk of peripheral artery occlusion disease (The correlation is comprehensively studied, which is not our interested issue) [13,14], to prevent misinterpretation, patients with hip fracture (ICD codes: 835, 9240, and 9280) were excluded from this research. Therefore, the term “lower leg fracture” in this study refers to only tibial, fibular, or tibiofibular fracture but not hip fracture, even it is a fracture located on a lower limb.

Patients with a history of SAET of the lower extremity before the index date or incomplete data were excluded in this study.

### 2.3. Survey of Confounding Factors of SAET

Comorbidities were also important confounding factors in the NHIRD study. We defined the comorbidities as diabetes (ICD-9-CM: 250) [15], hypertension (ICD-9-CM: 401–405) [16], and gout (ICD-9-CM: 274) [17], which recorded before the index date. All study subjects were followed until the subject is expired, withdrawn from the NHIRD program, had an onset of lower extremity SAET, or the endpoint date of this study, December 31.

All the 269,688 patients in the two cohorts are regrouped into two groups—with and without an onset of SAET after lower leg fracture. Demographics and percentages of having prior lower leg fractures and comorbidities mentioned above were recorded and analyzed.

### 2.4. Statistical Analysis

Propensity score matching for tibia and fibula fracture was applied to match the tibia and fibula fracture patients to non-fracture subjects one by one. Matching variables included gender, age, index year of tibia and fibula fracture, diabetes, hypertension, and gout. Logistic regression was used to estimate the incidence differences between the two cohorts. 

Patients with fracture of tibia and fibula were matched (1:1 ratio) with those who without fracture of tibia and fibula according to their propensity score, which consisted of age (every 5 years span), gender, index year, and comorbidities through nearest neighbor matching, initially to the eighth digit and then as required to the first digit. Therefore, matches were first made within a caliper width of 0.0000001, and then the caliper width was increased for unmatched cases to 0.1. We reconsidered the matching criteria and performed a rematch (greedy algorithm). For each patient with fracture of tibia and fibula, the corresponding comparisons (non-fracture of tibia and fibula) were selected based on the nearest propensity score.

Baseline demographics and comorbidities were calculated from the data from the cohort without fracture, while the difference between the two cohorts was tested by standardized mean difference (SMD) and indicated a negligible difference at SMD ≤ 0.1 [18]. The incidence rate of lower extremity SAET was calculated as the number of newly recorded lower extremity SAET divided by the following period (per 10,000 person-year). The Kaplan–Meier method was applied to calculate the cumulative incidence of lower extremity SAET, and the difference of two survival curves was tested by the log-rank test. Furthermore, the potential risk factors of lower extremity SAET and stratified analysis were conducted by the Cox proportional hazard model and presented by hazard ratio (HR), adjusted hazard ratio (aHR) (adjusted the demographic factors and comorbidities), and 95% confidence interval (95% CI). All statistical analyses were performed using SAS statistical software, v.9.4 (SAS Institute Inc., Cary, NC, USA). The cumulative incidence curves were plotted using R software, and the significant criteria were set at two side *p*-value less than 0.05.

## 3. Results

**Figure 1:**
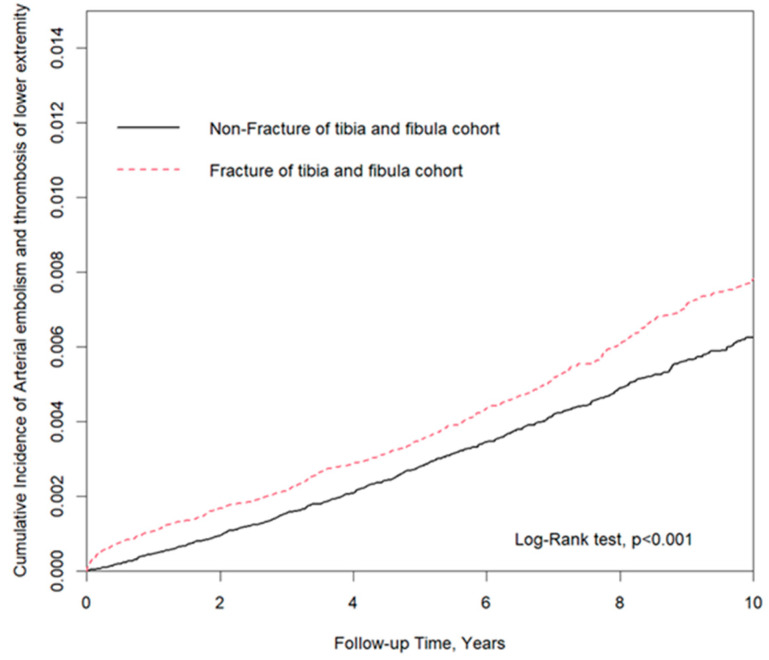
Kaplan–Meier analysis plot showed that, by the end of the ten-year follow-up period, the cumulative incidence of PAOD was significantly higher for the lower leg fracture cohort than for the non-fracture cohort (log-rank test: *p* < 0.001).

Figure 1 showed that, by the end of the ten-year follow-up period, the cumulative incidence of PAOD was significantly higher for the lower leg fracture cohort than for the non-fracture cohort (log-rank test: *p* < 0.001).

This study included 134,844 eligible patients with tibia and fibula fracture and 134,844 patients without tibia and fibula fracture (Table 1). Of the 134,844 patients with tibia and fibula fracture, 58.6% were male, and the mean onset age was approximately 48.2 years. Hypertension, diabetes, and gout, defined as comorbidities, showed an insignificant difference between the two cohorts (*p* > 0.05).

Table 2 presents potential risk factors of lower extremity SAET. Patients with tibia and fibula fracture (aHR = 1.39, 95% CI = 1.24–1.54), males (aHR = 1.53, 95% CI = 1.37–1.71), increased onset age (aHR = 3.97, 95% CI = 3.11–5.06; aHR = 10.96, 95% CI = 8.53–14.07), hypertension (aHR = 1.78, 95% CI = 1.55–2.03), diabetes (aHR = 5.57, 95% CI = 4.90–6.32), and gout (aHR = 1.43, 95% CI = 1.13–1.81) showed a significantly high risk of developing lower extremity SAET after being adjusted with demographic and comorbidity factors.

In Table 3, we conducted multivariable stratified analysis to confirm that tibia and fibula fracture increased the risk of lower extremity PAOD in females (aHR = 1.37, 95% CI = 1.16–1.62), males (aHR = 1.40, 95% CI = 1.122–1.61), ≤40 years (aHR = 3.08, 95% CI = 1.83–5.17), 40–64 years (aHR = 1.51, 95% CI = 1.26–1.79), ≥65 years (aHR = 1.22, 95% CI = 1.05–1.40), hypertension (aHR = 1.22, 95% CI = 1.02–1.46), diabetes mellitus (aHR = 1.24, 95% CI = 1.05–1.48) and gout (aHR = 1.65, 95% CI = 1.05–2.60).

When stratified by follow-up years and discussed gender separately, males (aHR = 1.82, 95% CI = 1.30–2.55) and females (aHR = 1.84, 95% CI = 1.38–2.47) who were followed up for less than two years from the index date showed a significantly higher risk of lower extremity SAET, respectively (Table 4).

## 4. Discussion

In our study, patients with SAET are significantly correlated to risk factors including lower leg fracture, male gender, elder age (>45 years), and comorbidities (hypertension, diabetes mellitus, and gout) (Table 2). The results of subgroup analysis show that patients with lower leg fractures suffered a higher SAET risk than the non-fracture group regardless of grouping by gender, age, or comorbidities (Table 3). The results mentioned above reveal that lower leg fracture is firmly correlated with SAET in either the general population or subunits population with different ages, gender, or comorbidities. Additionally, patients with lower leg fractures had a higher incidence rate of SAET within two years after injury (Table 4). Our study is the first study that clarified the correlation between lower leg fracture and SAET and the riskiest period of onset of SAET. Based on this study, an image survey (e.g., angiography) within two years might be considered for a patient with a lower leg fracture. 

As mentioned above, patients with lower leg fracture had a significant incidence rate of SAET in a ten-year follow-up (Figure 1), which may recommend the importance of routine vascular examinations after injury. A larger prospective study suggested that computed tomography angiography is a useful test in identifying early vascular injuries after trauma, and the incidence of vascular injury in open tibial fractures is 29% [19]. In addition, duplex ultrasonography or angiography was indicated for patients with orthopedic surgery or abnormal neurovascular examinations after blunt lower extremity trauma [20]. However, the values of these instruments on long-term follow-up of artery patency after injury were not well established. Therefore, further studies are necessary.

In this study, the SAET was associated significantly with many confounding factors such as lower leg fracture, male, increased age, and comorbidities (Table 2). After stratification with male, increased age, and comorbidities, the results suggested the fractured cohort had higher incidence of SAET than the non-fracture cohort (Table 3). These findings may imply lower leg fracture will seriously impact neighboring vessels; therefore, significant influences would be found in each subgroup.

Although patients with lower leg fracture in the three age levels (<45, 40–64, and ≥65 years) had significant risks of SAET compared with the non-fracture group (Table 3), the underlying mechanism of injury may differ. Vascular injuries in the younger age after fracture were usually associated with motor vehicles, especially motorcycle accidents, in which the most common injured vessel and vascular injury was the anterior tibial artery (36%) and complete arterial occlusion (62.7%), respectively [5,21]. In contrast, the low-energy fracture caused by a slip, trip, or fall from a standing pasture was the most common cause of lower-extremity fracture in the aged group [22]. Thus, the comorbidity factor of osteoporosis in aging-associated fractures should not be neglected [23]. 

Concerning the incidence and HR of SAET stratified by follow-up years, our results showed that there were 1.84- and 1.82-folds of SAET recurrence rates within two years in males and females, respectively, matching some findings that PAOD presents as tibial non-union in two years after injury [24,25,26]. However, there is no significant difference in SAET incidence later than two years of follow-up. A possible explanation may be spontaneous thrombolysis, revascularization [27], or re-establishment of the collateral circulation [28]. Further studies may be required to determine the comprehensive knowledge of the correlation between lower leg fracture and subsequent SAET.

With reliable diagnoses and a high follow-up rate, the study of SAET risks in patients with lower leg fracture is strengthened by the large population with available data regarding longitudinal assessments and subgroup analyses. However, certain limitations exist. Firstly, variables including lifestyle factors such as smoking, dietary habits, drinking, socioeconomic status, drug use, and genetic factors were not obtained to adjust the risk of developing SAET because of databank limitation. Secondly, the diagnoses of SAET were based on admission diagnoses and codes; thus, precise analysis and severity/grades were lacking. Thirdly, because the data used were anonymous, relevant clinical variables, such as severity or type of fracture, physical activity, injury mechanisms, operation findings, imaging results, and laboratory data, were not available. At last, biases from retrospective nature should also be noted. Nonetheless, the results generated from NHIRD regarding lower leg fracture, surgery, and SAET diagnosis were highly reliable. The numerous databases with comprehensive statistical analysis are helpful to diminish some of the biases mentioned above. 

Summarily, our findings demonstrated that patients with lower leg fractures had a higher risk of SAET than the non-fracture group, and the high incidence rate lasted for two years after injury. However, based on the retrospective nature and limited study number, further studies are warranted.

## Figures and Tables

**Table 1:** Demographic characteristics and comorbidities of patients newly diagnosed fracture of tibia and fibula in Taiwan during 2000–2012.

Characteristics	Total	Fracture of Tibia and Fibula		*p*-Value
		No *n* = 134,844	Yes *n* = 134,844	
**Gender**				0.98
Female	111,724	55,859 (41.4)	55,865 (41.4)	
Male	157,964	78,985 (58.6)	78,979 (58.6)	
**Age**				>0.99
<40	93,997	46,995 (34.9)	47,002 (34.9)	
40-64	124,507	62,251 (46.2)	62,256 (46.2)	
≥65	51,184	25,598 (19)	25,586 (19)	
**Mean (SD) ^a^**		48.2 (17.1)	48.2 (17.1)	>0.99
**Baseline Comorbidity**				
Hypertension	23,526	11,765 (8.7)	11,761 (8.7)	0.98
Diabetes mellitus	17,180	8583 (6.4)	8597 (6.4)	0.91
Gout	4733	2362 (1.8)	2371 (1.8)	0.90

Chi-square test, ^a^ *t*-test.

**Table 2:** Cox model measured hazard ratio and 95% confidence intervals of arterial embolism and thrombosis of lower extremity associated with and without fracture of tibia and fibula patients.

Characteristics	Event	Crude		Adjusted	
	(*n* = 1360)	HR (95% CI)	*p*-Value	HR (95% CI)	*p*-Value
**Fracture of Tibia and Fibula**					
No	593	Ref.		Ref.	
Yes	767	1.32 (1.19–1.47)	<0.001	1.39 (1.24–1.54)	<0.001
**Gender**					
Female	559	Ref.		Ref.	
Male	801	1.00 (0.90–1.11)	1.000	1.53 (1.37–1.71)	<0.001
**Age at Baseline**					
<45	77	Ref.		Ref.	
45–64	516	5.48 (4.31–6.96)	<0.001	3.97 (3.11–5.06)	<0.001
≥65	767	25.49 (20.16–32.23)	<0.001	10.96 (8.53–14.07)	<0.001
**Baseline Comorbidity**					
Hypertension	480	8.67 (7.75–9.71)	<0.001	1.78 (1.55–2.03)	<0.001
Diabetes mellitus	522	13.87 (12.42–15.49)	<0.001	5.57 (4.90–6.32)	<0.001
Gout	78	4.64 (3.69–5.83)	<0.001	1.43 (1.13–1.81)	0.003

Abbreviation: HR, hazard ratio; CI, confidence interval; Adjusted HR: adjusted for gender, age, and comorbidities in Cox proportional hazards regression.

**Table 3:** Incidence rates, hazard ratio and confidence intervals of arterial embolism and thrombosis of lower extremity in different stratification.

Variables	Matched Cohort			Fracture of Tibia and Fibula			HR			
	*n* = 134,844			*n* = 134,844			Crude	*p*-Value	Adjusted	*p*-Value
	Event	Person Years	IR	Event	Person Years	IR	(95% CI)		(95% CI)	
**Overall**	593	964,777	6.15	767	945,131	8.12	1.32 (1.19–1.47)	<0.001	1.39 (1.24–1.54)	<0.001
**Gender**										
Female	249	396,001	6.29	310	389,968	7.95	1.27 (1.07–1.50)	0.006	1.37 (1.16–1.62)	0.0002
Male	344	568,776	6.05	457	555,164	8.23	1.36 (1.19–1.57)	<0.001	1.40 (1.22–1.61)	<0.001
**Age at Baseline**										
<40	19	359,942	0.53	58	359,025	1.62	3.07 (1.83–5.15)	<0.001	3.08 (1.83–5.17)	<0.001
40–64	213	451,823	4.71	303	442,063	6.85	1.46 (1.23–1.74)	<0.001	1.51 (1.26–1.79)	<0.001
≥65	361	153,012	23.59	406	144,044	28.19	1.20 (1.04–1.38)	0.011	1.22 (1.05–1.40)	0.007
**Baseline Comorbidity**										
Hypertension	226	61,834	36.55	254	58,386	43.50	1.19 (1.00–1.43)	0.055	1.22 (1.02–1.46)	0.031
Diabetes Mellitus	245	45,501.9	53.84	277	42,334	65.43	1.23 (1.04–1.46)	0.019	1.24 (1.05–1.48)	0.013
Gout	31	13,022.4	23.81	47	12,515	37.55	1.59 (1.01–2.49)	0.047	1.65 (1.05–2.60)	0.030

Abbreviation: IR, incidence rates, per 10,000 person-years; HR, hazard ratio; CI, confidence interval; Adjusted HR: adjusted for gender, age, and comorbidities in Cox proportional hazards regression.

**Table 4:** Incidence rates, hazard ratio and confidence intervals of arterial embolism and thrombosis of lower extremity in different stratification.

Variables	Matched Cohort			Fracture of Tibia and Fibula			HR			
	*n* = 134,844			*n* = 134,844			Crude	*p*-Value	Adjusted	*p*-Value
	Event	Person Years	IR	Event	Person Years	IR	(95% CI)		(95% CI)	
**Female**										
**Follow-up Years**										
<2	53	107,846	4.91	92	106,826	8.61	1.75 (1.25–2.46)	0.001	1.82 (1.30–2.55)	0.001
2–5	86	129,404	6.65	106	184,431	5.75	0.86 (0.63–1.18)	0.345	0.93 (0.68–1.28)	0.664
≥5	110	158,751	6.93	145	155,450	9.33	1.35 (0.97–1.73)	0.078	1.50 (0.92–1.93)	0.101
**Male**										
**Follow-up Years**										
<2	70	152,339	4.60	125	150,228	8.32	1.81 (1.35–2.42)	<0.001	1.84 (1.38–2.47)	<0.001
2–5	73	127,692	5.72	114	180,974	6.30	1.10 (0.84–1.43)	0.495	1.12 (0.86–1.45)	0.421
≥5	168	232,007	7.24	218	223,962	9.73	1.35 (0.98–1.65)	0.094	1.50 (1.00–1.93)	0.051

Abbreviation: IR, incidence rates, per 10,000 person-years; HR, hazard ratio; CI, confidence interval; Adjusted HR: adjusted for gender, age, and comorbidities in Cox proportional hazards regression.

## Data Availability

This study used inpatient claims data from the Taiwan National Health Insurance Research Database (NHIRD). This database contains detailed medical histories of the hospitalized enrollees in Taiwan. Based on the guideline of Taiwan Ministry of Health and Welfare (TMHW), only citizens of the Taiwan are eligible to apply the NHIRD for research projects (https://nhird.nhri.org.tw/en/Data_Protection.html (accessed on 17 September 2019)). The database we applied is only limited to our research purpose. All applicants must follow the Computer-Processed Personal Data Protection Law and related regulations of National Health Insurance Administration and NHRI (http://www.winklerpartners.com/?p=987 (accessed on 17 September 2019)). The ownership of NHIRD is belong to TMHW and the right to use is belong to the researchers. However, other researchers are able to request data access following the regulations of TMHW.
